# Medical students’ knowledge and attitude towards using artificial intelligence in medical education and practice: a pre-post study

**DOI:** 10.1186/s12909-026-09677-8

**Published:** 2026-06-23

**Authors:** Heba Tarek Emara, Mohamed Azmy Khafagy, Nermeen Ahmed Niazy, Sherehan Adel Abdel-Salam

**Affiliations:** Public Health and Community Medicine Department, Faculty of Medicine, Mansoura University, Mansoura, 35516 Egypt

**Keywords:** Medical students, Knowledge, Attitude, Artificial intelligence, Medical education, Medical practice

## Abstract

**Background:**

This study aims to evaluate the knowledge and attitudes of Mansoura medical students towards artificial intelligence (AI) use in medical education and practice before and after an educational intervention as to the best of authors’ knowledge, all studies conducted in Egypt in this concern are observational.

**Methods:**

This is a Pre-post study conducted in the Faculty of Medicine, Mansoura University, Egypt, during the academic year 2024–2025. Study participants were medical students enrolled in the integrated modular-based education. Two hundred and twenty students completed the questionnaires before the intervention; however, only 189 of them completed the questionnaires after the intervention.

**Results:**

Only 15.9% of students had previous education about AI during medical study. The most frequent source of previous education was social media platforms as well as AI courses (10.6%). Unfortunately, the least frequent sources were undergraduate curriculum (1.1%) and literature reviews as well as published research articles (0.5%). More than half of students had moderate knowledge and attitude towards AI use in medical education (53.4%) before the intervention, while after intervention, more than half of students had good knowledge and attitude (50.8%). Poor knowledge and attitude decreased from 17.3% to 8.5% after intervention. Regarding medical practice, good knowledge and attitude increased from 17.5% to 36.5% after the intervention. Poor knowledge and attitude decreased from 13.8% to only 5.3% after the intervention with statistically significant difference between the results before and after the intervention (*p* < 0.001).

**Conclusion:**

The intervention was associated with improved students’ knowledge and attitude towards AI use in both medical education and practice. The intervention was also associated with an increase in the familiarity of medical students with the different AI tools used in both medical education and practice. The intervention was also associated with an increase in the passion of students to learn more. However, it had less impact on increasing their confidence in their ability to use AI or willing to be operated upon by an AI machine. Therefore, students need a more comprehensive program to increase their confidence.

**Supplementary Information:**

The online version contains supplementary material available at 10.1186/s12909-026-09677-8.

## Background

Artificial intelligence (AI) is currently one of the trending topics among technological advancements [1]. It has the capacity to affect the field of healthcare significantly [2]. In medicine, AI applications aim to “support decision-based medical tasks through knowledge- and/or data-intensive computer-based solutions that ultimately support and improve the performance of a human care provider” [3]. Various functions can be performed by AI systems to help healthcare providers in several medical fields as personalized medicine, monitoring of health, management of health data, disease diagnostics, development of drugs, and analyzing health plans as well as medical and surgical treatments [4].

Integrating AI in medical curriculums has many advantages for students. It can provide feedback about performance of students helping to monitor their improvement level. It represents a stimulating and multisensory environment increasing engagement and attention of students, leading to improving knowledge acquisition and gaining competencies. It also has the potential to engage students in problem-based learning with various cases at any time. It helps observing medical cases in different grades, providing various interventions in a safe environment [5].

Therefore, medical education has to catch attention as medical students represents the future doctors who need to have appropriate advanced clinical practices, which can be done with the help of AI applications, which need new competencies to utilize a huge health dataset, analyze, and report outcomes [6].

Although high-income countries make significant investments in the research of AI in healthcare, developing countries have been facing challenges in research, education, and implementation of AI. On the other hand, limited resources urge the necessity of the knowledge about AI to reduce diagnostic errors and workload [7]. Regardless of these challenges, the use of AI in healthcare is promising, as it helps to address the limitations of traditional diagnostic and treatment methods, and reduce patient anxiety as well as medical errors. However, misconceptions exist about AI which influence healthcare [8–13]. Medical students have been reported to not have much fear of being replaced by AI as doctors [14]. However, some students may have anxiety due to the probability of being displaced. Therefore, they may be discouraged to consider certain specialties [15].

There is not enough discussion about AI in today’s medical education [16]. Moreover, to the best of authors’ knowledge, all studies conducted in Egypt and published in this concern are observational. Therefore, this study aims to evaluate the knowledge and attitudes of integrated modular-based medical students, at Mansoura University, Mansoura, Egypt toward AI use in medical education and practice before and after an educational intervention. Therefore, if this intervention proves to be useful, it can be recommended to be generalized to all medical students.

## Methods

This is a pre-post study: before and after an educational intervention, conducted in the Faculty of Medicine, Mansoura University, Mansoura, Egypt, during the academic year 2024–2025.

Study participants were medical students enrolled in the integrated modular-based education. Inclusion criteria included all students enrolled in the second year as a representative sample for the junior students and all students enrolled in the second internship year as a representative sample for the senior students.

### Sample size calculation and technique

Sample size was calculated using the MedCalc program version 19 applying the paired samples T test with a mean difference for knowledge and attitude of students about using AI in medical practice between pre and post intervention of 2.1777 and standard deviations (SD) of 5.53 and 6.11 respectively (derived from the pilot study), with a power of 0.90 and a two-sided alpha error of 0.05. Therefore, the calculated sample size was 77 student pairs (pre and post). This was multiplied by 2 to compensate for the design effect of the stratified sampling technique employed. Therefore, the sample size was 154 student pairs (pre and post). Adding 20% for attrition, the estimated sample size was a total of 185 students at least.

Medical students enrolled in the second year and the second internship year were recruited using a stratified cluster sampling method. Within each year, total number of students is normally divided into groups of average 30 students. Total sample was selected from the 2 years, with each year considered a stratum with proportional allocation according to the number of students in each year. Within each stratum, a number of groups (clusters) was randomly selected using computer generated random tables using SPSS (Statistical Package for Social Sciences Inc., version 25). All students in the selected clusters were chosen. Self-administered questionnaires were distributed before and after the educational intervention after getting permission from the heads of departments. The after-intervention questionnaires were distributed two to three weeks after the intervention. Two hundred and twenty students completed the questionnaires before the intervention; however, only 189 of them completed the questionnaires after the intervention. Thus, response rate was 85.9%; therefore, the loss of follow up rate was 14.1%. Baseline characteristics were compared between those who lost follow-up and those who were followed-up to ensure avoidance of attrition bias. There was no significant difference between them regarding all sociodemographic characteristics, having previous education about AI, thinking that learning programming or mathematics would help to comprehend the principles of AI, hearing about every tool mentioned for medical education and practice, and preferring every method for learning mentioned. All other variables regarding knowledge and attitude of both medical education and practice statements as well as preintervention scores showed non-significant difference between them.

### Study tools

#### Before-intervention questionnaire

Students filled a structured self-administered English questionnaire to collect information about four sections. The first section was about the students’ socio-demographic characteristics such as age, sex, academic year, accommodation, family income, and exercise. Students were told to write their phone numbers and E-mails accurately to be used for joining the data before and after the intervention. The second section comprised general questions about technology background and use of AI in medicine which was derived from previous studies [17–20]. The third section was about knowledge and attitude towards AI and its application in medical education, which included 15 statements, which was adapted from previous research [19]. To maintain structural integrity and validity of the original questionnaire, we utilized the scoring system exactly as prescribed by the original reference to allow direct comparing of our findings to prior studies using the same metric. Each statement was scored on a scale of 1 to 5, with 1 being strongly disagree and 5 being strongly agree. This score was reversed for three statements (statements 8, 14, and 15). The total score for medical students’ knowledge and attitude was computed by summation of the 15 statements, then it was categorized by considering < 37 score as a poor level, 38–57 moderate, and > 57 good [19]. The fourth section was about knowledge and attitude towards AI and its use in medical practice, which involved seven statements. Each statement was scored on a scale of 1 to 5, with 1 being strongly disagree and 5 being strongly agree. This score was reversed for two statements (statements 5 and 7). The overall score for medical practice was computed by summation of the seven statements, then it was classified by considering < 17 score as a poor level, 18–27 moderate, and > 27 good [19].

#### Educational intervention

The intervention was an attractive concise video of about a fifteen-minute duration. The video was a recorded PowerPoint presentation (Microsoft PowerPoint 365) with the voice of the main investigator in a combined English and Arabic language for increasing the clarity, attention, and engagement of students. The design of the educational video was grounded in Mayer’s Cognitive Theory of Multimedia Learning by integrating interactive engaging contents such as images, videos, online websites to reduce the cognitive load, increase the attention and decrease the loss of concentration of students. The video length was also kept as short as possible (by trimming and increasing the speed of the attached videos) dividing it into clear bite-sized segments and utilizing signaling (only key text) as well as weeding (removing unnecessary objects or complex background) to align with the Cognitive Load Theory to ensure that the presented information did not exceed the capacity of the working memory. The principal investigator got great feedbacks from the participants directly after watching the intervention. The educational content included the items tested in the questionnaires. The learning objectives included but (not exclusive):

- to demonstrate the different AI tools used in medical education and practice in a practical manner with examples for each tool as personalized learning platforms, gamification tools, ……….etc,
- to describe importance of (although not essential) programming and mathematics to apply AI in medicine,
- to discuss the ethical implication of AI in education,
- to explain how academic grading is enhanced by AI,
- to describe the AI role in revolutionizing learning methods,
- to demonstrate clinical decision support systems as UpToDate website,
- to summarize how AI support radiologists by medical imaging analysis,
- to distinguish between different types of AI chatbots in healthcare as administrative, diagnostic, mental health support, etc…,
- to demonstrate the concept of robotic surgery,
- to discuss AI literacy importance for healthcare providers,
- to explain why AI cannot replace human teachers or doctors.
  For the purpose of replication, a pdf version of the educational content (without sound or active videos) and a link of the recorded video are available on reasonable request from the corresponding author.

#### The after-intervention questionnaire

The after-intervention questionnaires were distributed two to three weeks after the intervention. Students were told again to write their phone numbers and E-mails accurately to be used for joining the data before and after the intervention. The after-intervention questionnaire was the same as the before-intervention questionnaire but without the socio-demographic characteristics and technology background.

#### Pilot testing of the questionnaire

An external pilot study was conducted to assess clarity, feasibility, and internal consistency of the questionnaire among 19 students. The Cronbach’s alpha coefficient was 0.910 for knowledge and attitude of students about using AI in medical education and 0.749 for knowledge and attitude of students about using AI in medical practice; respectively. This was followed by another external pilot study of 30 students to calculate the sample size.

### Ethical considerations

Consent was obtained from the authority of the Faculty of Medicine, Mansoura university before the conduction phase of the study. Ethical approval from Institutional Research Board of Mansoura Faculty of Medicine (MFM-IRB) was obtained (reference number: MD.24.10.912). Written informed consent was obtained from the students who were assured of their anonymity and the confidentiality of their responses. All methods were conducted in accordance to the Declaration of Helsinki.

### Statistical analysis

The internal consistency of the questionnaire scales was examined by using Cronbach α coefficients during the pilot study. The completed questionnaires were subjected to revision, and the collected data were coded, processed and analyzed through SPSS (Statistical Package for Social Sciences Inc., version 25). A descriptive analysis of the collected data was performed in the form of frequencies and percentage for qualitative data and mean ± SD or median (Q1-Q3) for quantitative data. For comparison of the pre and post intervention results, paired T-test for normally distributed continuous variables and McNemar test for categorical variables were used. For quantitative variables, Student T-test was used to compare the means between different groups. The paired analyses used only matched pre-post responses, meaning only students who completed both the pre- and post-intervention questionnaires were included. Effect size is reported as percent of change which equals $\frac{post\:agreement\:percent-pre\:agreement\:percent}{pre\:agreement\:percent}$. Pearson correlation was used to assess the correlation between normally distributed continuous variables. Statistical tests done were two-tailed and the level of statistical significance chosen was 0.05. Multiple bar charts were used to visualize the relationship between qualitative variables.

## Results

Out of 189 students, there was nearly equal sex distribution with slightly higher proportion of males than females (54.3% vs. 49.7% respectively). The mean age of them was 21.5 years with SD of 2.6 years. Distribution of students over academic years was more over the second year (64.6%) than the second internship year (35.4%). Total family income was enough among 80.4% of students. About 79% of students lived with their families or friends, while 44% of students never exercised (Table 1).

**Table 1:** Demographic characteristics of the studied students (*n* = 189)

Variables		Mean	SD
Age in years		21.5	2.6

**Variables**		**n**	**%**
Sex	Female	94	49.7%
	Male	95	50.3%
Academic year	Second year	122	64.6%
	Second internship year	67	35.4%
Total family income	Not enough (barely routine expenses)	37	19.6%
	Enough (routine and emergency expenses/ save money)	152	80.4%
Accommodation	Alone	39	20.6%
	With family/ friends	150	79.3%
The frequency of doing exercise per week	Never	83	43.9%
	One to two times	56	29.6%
	Three times or more	50	26.5%

Data were presented as number (*n*) and percent (%) or mean and standard deviation (SD)

Only 15.9% of students had previous education about AI during medical study. Among those who had previous education, the most frequent source of education was social media platforms as well as AI courses (10.6%), followed by colleagues/friends/professors (7.9%). Unfortunately, the least frequent sources were undergraduate curriculum (1.1%) and literature reviews as well as published research articles (0.5%). More than half of students spent 3 to 6 h per day on their mobile phones. About 69% reported that most of this time was spent in entertainment, while only 2.6% most of this time in taking courses, and 61% reported studying through iPad or tablets (Table 2).

**Table 2:** History of previous education about AI in medicine and technology use in the studied students before the intervention (*n* = 189)

		n	%
Having previous education about AI during medical study	No	159	84.1%
	Yes	30	15.9%
If yes, source of education about AI during medical education/training^#^	Undergraduate curriculum	2	1.1%
	Elective courses provided by the university	4	2.1%
	AI courses	20	10.6%
	Research project	10	5.3%
	Social media platforms	20	10.6%
	Literature reviews and published research articles	1	0.5%
	Colleagues/friends/professors	15	7.9%
	Scientific events (conferences, workshops, …etc.)	4	2.1%
Time spent on mobile phone	Less than 3 h per day	26	13.8%
	Between 3 till 6 h per day	96	50.8%
	More than 6 h per day	67	35.4%
Most of the time spent on phone is in	Entertainment	131	69.3%
	Studying	53	28.0%
	Courses	5	2.6%
Study through iPad or Tablets	No	74	39.2%
	Yes	115	60.8%

Data were presented as number (*n*) and percent (%)

*AI* Artificial intelligence

^#^Responses are non-mutually exclusive

The most preferred format for learning about AI in medicine before intervention was workshops (67.2%), followed by small group discussion (64%). The least preferred format was conferences (53.4%).

After the intervention, proportion of students preferring small group discussion increased to be equal to that of workshops (82%), where both were the most frequent preferred formats. Generally, proportion of students preferring all formats showed statistically significant increase after the intervention with the highest increase for collaborative activities with other departments (mathematics, computer science) (+ 28.3%, *p* = 0.001), followed by small group discussion (+ 28.1%, *p* < 0.001), then extracurricular activities as field visits, presentations, writing essays or reports, case study, journal clubs (+ 27.5%, *p* = 0.001). The lowest increase was for lectures (+ 18.9%, *p* = 0.010) (Table 3).

**Table 3:** Preferred format for learning about artificial intelligence in medicine from the studied students’ point of view before and after intervention (*n* = 189)

The preferred format for learning about artificial intelligence in medicine^#^	Before intervention		After intervention		Percent of change	*P*-value
	*n*	%	*n*	%		
Lectures	111	58.7%	132	69.8%	+ 18.9%	**0.010**
Small group discussion	121	64.0%	155	82.0%	+ 28.1%	**< 0.001**
Conferences	101	53.4%	125	66.1%	+ 23.8%	**0.005**
Workshops	127	67.2%	155	82.0%	+ 22.0%	**< 0.001**
Extracurricular activities*	109	57.7%	139	73.5%	+ 27.5%	**0.001**
Collaborative activities with other departments (mathematics, computer science)	113	59.8%	145	76.7%	+ 28.3%	**0.001**

Data were presented as number (*n*) and percent (%) or mean and standard deviation. McNemar test was used

Bold *p*-values are significant at level < 0.05

*Examples of extracurricular activities are field visits, presentations, writing essays or reports, case study, journal clubs

^#^Responses are non-mutually exclusive

**Fig. 1:**
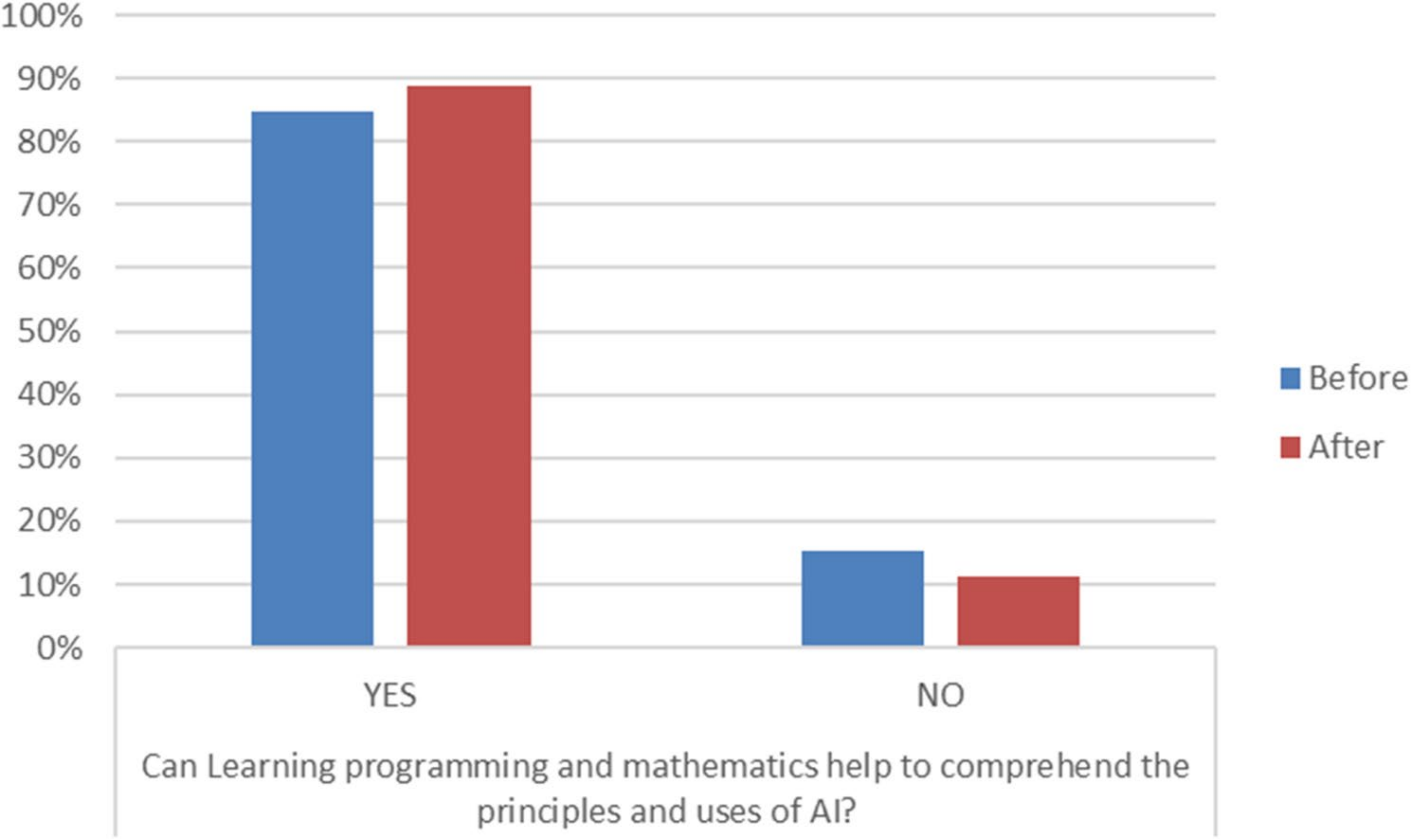
Comparison of students’ perception of the importance of learning programing and mathematics to comprehend the principles and uses of artificial intelligence (AI) before and after the intervention (*n* = 189)

The percent of students reporting the importance of learning programing and mathematics to comprehend the principles and uses of AI before the intervention was 84.7% which increased to 88.9% after the intervention with an improvement of + 5% with no significant difference (*p* = 0.195) (Fig. 1).

The most frequent AI tool in medical education students previously heard about before intervention was chatbots for student support (57.7%), followed by automated exam grading systems (47.6%). The least frequent tool was gamification tools (20%). After the intervention, proportion of students previously hearing about interactive smartboards and automated exam grading systems increased to be equal to that of chatbots for student support (86.2%), which were the most frequent tools students heard about. The least frequent tool was gamification tools (69.3%). Generally, proportion of students who heard about all tools showed statistically significant increase after the intervention with the highest increase for gamification tools (235.9%, *p* < 0.001), followed by virtual patient learning (+ 137.9%, *p* < 0.001), then virtual reality simulation (+ 100%, *p* < 0.001). The lowest increase was for chatbots for student support (+ 49.5%, *p* = 0.010).

The most frequent AI tool in medical practice students previously heard about before intervention was electronic health records (51.3%), followed by robotic surgery (48.7%). The least frequent tool was virtual assistants (32.8%). After the intervention, the most frequent AI tool students previously hearing about was medical imaging analysis (84.7%), followed by robotic surgery (84.1%). The least frequent tool was predictive analytics (69.8%). Generally, proportion of students who heard about all tools showed statistically significant increase after the intervention with the highest increase for Virtual assistants (+ 124.1%) (Table 4).

**Table 4:** The AI tools which can be used in medical education and practice about which the studied students previously heard before and after intervention (*n* = 189)

AI tools in medical education^#^	Before intervention		After intervention		Percent of change	*P*-value
	*n*	%	*n*	%		
Personalized learning platforms	83	43.9%	158	83.6%	+ 90.4%	**< 0.001**
Virtual patient learning	66	34.9%	157	83.1%	+ 137.9%	**< 0.001**
Gamification tools	39	20.6%	131	69.3%	+ 235.9%	**< 0.001**
Chatbots for student support	109	57.7%	163	86.2%	+ 49.5%	**< 0.001**
Interactive smartboards	84	44.4%	163	86.2%	+ 94.0%	**< 0.001**
Virtual reality simulation	75	39.7%	150	79.4%	+ 100.0%	**< 0.001**
Automated exam grading systems	90	47.6%	163	86.2%	+ 81.1%	**< 0.001**

AI tools in medical practice^#^	Before intervention		After intervention		Percent of change	*P*-value
	n	%	n	%		
Electronic health records	97	51.3%	155	82.0%	+ 59.8%	**< 0.001**
Clinical decision support system	66	34.9%	146	77.2%	+ 121.2%	**< 0.001**
Medical imaging analysis	91	48.1%	160	84.7%	+ 76.1%	**< 0.001**
Personalized medicine	70	37.0%	135	71.4%	+ 93.0%	**< 0.001**
Virtual assistants	62	32.8%	139	73.5%	+ 124.1%	**< 0.001**
Robotic surgery	92	48.7%	159	84.1%	+ 72.7%	**< 0.001**
Predictive analytics	66	34.9%	132	69.8%	+ 100.0%	**< 0.001**

Data were presented as number (*n*) and percent (%). McNemar test was used

*AI* Artificial intelligence

Bold *p*-values are significant at level < 0.05

^#^ Responses are non-mutually exclusive

**Fig. 2:**
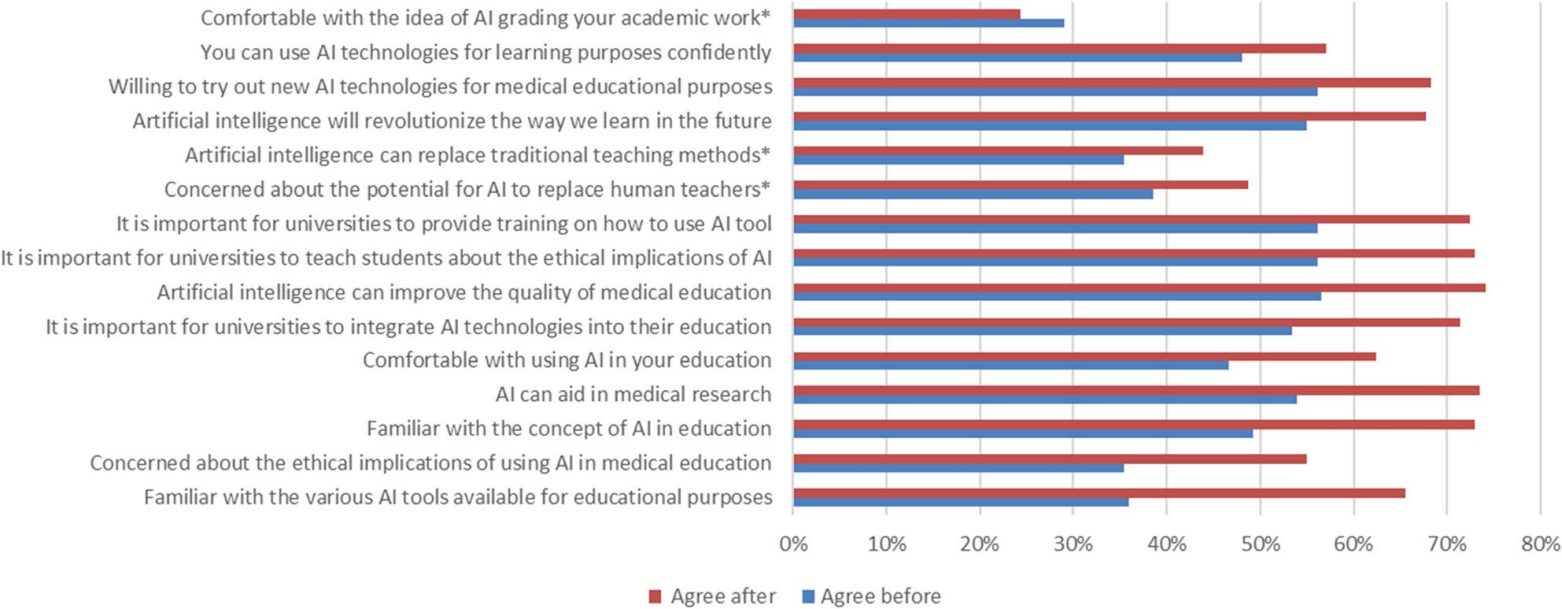
Comparison of percent of agreement regarding knowledge and attitude in medical education before and after the intervention among the studied students (*n* = 189). *Percent of disagreement was reported as the coding was reversed for these items. Variables are sorted in a descending order regarding percent of change. AI= Artificial intelligence

Regarding students’ knowledge and attitude towards AI and its application in medical education before the intervention, the statement with the highest agreement percent was “AI can improve the quality of medical education” (56.6% which increased to 74.1% after the intervention). The statement with the highest improvement of agreement percent after intervention was “Familiar with the various AI tools available for educational purposes” (+ 82.2%). While the statement with the lowest improvement of agreement percent was “You can use AI technologies for learning purposes confidently” (+ 18.7%). Percent of disagreement decreased after the intervention for the statement “Comfortable with the idea of AI grading your academic work” (-16.5%) (Fig. 2).

**Fig. 3:**
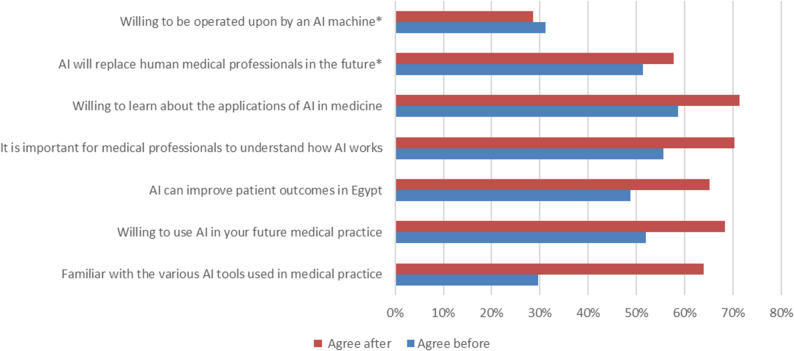
Comparison of the studied students’ percent of agreement regarding knowledge and attitude in medical practice before and after the intervention (*n* = 189). *Percent of disagreement was reported as the coding was reversed for these items. Variables are sorted in a descending order regarding percent of change. AI= Artificial intelligence

Regarding students’ knowledge and attitude towards AI and its application in medical practice before the intervention, the statement with the highest agreement percent was “Willing to learn about the applications of AI in medicine” (58.7% which increased to 71.4% after the intervention). The statement with the highest improvement of agreement percent after intervention was “Familiar with the various AI tools used in medical practice” (+ 116.2%), while the statement with the lowest improvement of agreement percent was “Willing to learn about the applications of AI in medicine” (+ 21.6%). Percent of disagreement decreased after the intervention for the statement “Willing to be operated upon by an AI machine” (-8.3%) (Fig. 3).

Scoring of knowledge and attitude in medical education and practice before and after the intervention among the studied students was described in Table 5. as Mean (SD), Median (Q1-Q3), and numbers as well as percentages after they were also recoded. Percent of change was also reported in the table.

**Table 5:** Scoring of knowledge and attitude in medical education and practice before and after the intervention among the studied students (*n* = 189)

Knowledge and attitude in medical education	Before intervention		After intervention			Percent of change	*P*-value
	Mean (SD)	Median (Q1-Q3)	Mean (SD)		Median (Q1-Q3)		
Total knowledge and attitude score	49.4(12.8)	50(45–60)	55.4(10.5)		58(50–63)	+ 12.2%	**< 0.001** ^a^
	**n**	**%**	**n**		**%**		
Poor (< 37)	33	17.5%	16		8.5%	-51.4%	**< 0.001** ^b^
Moderate (37–57)	101	53.4%	77		40.7%	-23.8%	
Good (> 57)	55	29.1%	96		50.8%	+ 74.6%	

Knowledge and attitude in medical practice	**Mean (SD)**	**Median (Q1-Q3)**	**Mean (SD)**	**Median (Q1-Q3)**		**Percent of change**	**P-value**
Total knowledge and attitude score	22.1(5.2)	22(20–26)	25.6(4.7)	26(23–29)		+ 15.8%	**< 0.001** ^a^
	**n**	**%**	**n**	**%**			
Poor (< 17)	26	13.8%	10	5.3%		-61.6%	**< 0.001** ^b^
Moderate (17–27)	130	68.8%	110	58.2%		-15.4%	
Good (> 27)	33	17.5%	69	36.5%		+ 108.6%	

Data were presented as mean (SD) or median (Q1-Q3)

*SD* Standard Deviation, *Q1* Quartile 1, *Q3* Quartile 3

Bold p-values are significant at level < 0.05

^a^ Paired t test was used

^b^ McNemar test was used

There was a strong positive significant correlation between medical students’ knowledge and attitude score in medical education and their score in medical practice both before and after the intervention. Moreover, the correlation was stronger after the intervention than before the intervention (*r* = 0.764, *p* < 0.001 vs. *r* = 0.898, *p* < 0.001; respectively).

## Discussion

The findings of the current study showed that the intervention was associated with improved students’ knowledge and attitude towards AI and its application in both medical education and practice. More than half of students had moderate knowledge and attitude towards AI and its application in medical education (53.4%) before the intervention, while after intervention, more than half of students had good knowledge and attitude (50.8%). Poor knowledge and attitude decreased from 17.3% before intervention to 8.5% after intervention. Regarding medical practice, good knowledge and attitude increased from 17.5% before the intervention to 36.5% after the intervention. Poor knowledge and attitude decreased from 13.8% before intervention to only 5.3% after the intervention with statistically significant difference between the results before and after the intervention.

Before conducting this study, all published studies in this concern were observational. However, recently, few studies have been published about assessing the effect of AI integration in medical curriculum. Two recent studies in 2024 and 2025 offering elective courses about AI found that the intervention was feasible, highly accepted, and beneficial for students with the former study reporting students’ satisfaction with the course [21, 22]. Another recent study by Hopson et al. introduced an educational intervention among premedical undergraduate showed that the intervention significantly improved participants’ knowledge and understanding of AI application in medicine [23].

Only 15.9% of participating students had previous education about AI during medical study. Among those who had previous education, the most frequent source of education was social media platforms and AI courses (10.6%). Unfortunately, the least frequent sources were undergraduate curriculum and literature reviews as well as published research articles. Similarly, previous studies reported that 15% and 23% had formal AI education before or during medical study [18, 20]. Social media was also reported by 58%, 34% of participants as the most frequent source for AI information among medical students in previous two studies [16, 18].

Despite the presence of various courses about AI that are offered online on the different learning platforms, only 10.6% reported being involved in AI courses before, although 61% reported studying through iPad or tablets and more than half of the students spent 3 to 6 h per day on their mobile phones; however, 69% reported that most of this time was spent in entertainment and only 2.6% spent most of this time in taking courses. May be, the reason for this is that students are busy studying the main curriculum courses for their medical study to obtain high scores in the final exams. A recent scoping review reported standardized framework lack for AI curriculum. It also reported the presence of global discrepancies. It reported the importance of digital competence, collaborative learning, and ethical training [24]. A recent meta-analysis indicated the importance of integrating educational courses in the curriculum. Moreover, it reported that students’ attitude was positive which expects the AI technology acceptance. On the other hand, AI ethics education and human-AI cooperation aspects should be dealt with [25]. Studies done in Egypt, India, and Canada reported that 75%, 46.8%, and 67% of participants; respectively agreed to integrate AI in medical curricula [20, 26, 27]. Another study reported the students’ need to involve AI training in medical curricula [18]. Moreover, it was reported that integrating AI in already existing courses may decrease curricular overload [28].

The most preferred format for learning about AI in medicine before intervention was workshops (67.2%), followed by small group discussion (64%). After the intervention, proportion of students preferring small group discussion increased to be equal to that of workshops (82%), where both were the most frequent preferred formats. Generally, proportion of students preferring all formats showed statistically significant increase after the intervention with the highest increase for collaborative activities with other departments (mathematics, computer science), followed by small group discussion, then extracurricular activities as field visits, presentations, writing essays or reports, case study, journal clubs. The lowest increase was for lectures. Similarly, an Indian study reported that 45% of participants preferred workshops followed by lectures (31%) [27]. The most frequent favorite method for learning in another previous study was workshops (30.4%), followed by extracurricular activities (29.8%) [18]. Workshops provide hands-on experience with practical application and direct interaction with AI tools. Current learners are different from previous learners. They are digital learners who are connected through the internet as they were growing up [29–31]. Small group discussions provide an environment for active participation, problem-solving, critical thinking, and real-world scenarios [32, 33]. University of Toronto and Queen’s university in the United Kingdom offer AI courses [34] using small group discussion, interactive lectures, electives, and e-modules as methods for learning [28].

Before the intervention, 84.7% of students recognized the importance of learning programing and mathematics to understand the principles of AI, and this increased slightly to 88.9% after the intervention with no significant difference. Jebreen et al. reported that 69% of students thought that learning programming and mathematics would help in comprehending AI principles [18]. In the United States, the Carle Illinois college of medicine has a curriculum emphasizing on mathematics and data science [34]. Learning information technology as programming is not a requirement for being prepared for AI. However, to have sufficient knowledge about basic and clinical medicine, biostatistics, data science, and evidence-based medicine is a requirement [35].

The most frequent AI tool in medical education students previously heard about before the intervention was chatbots for student support (57.7%), while the least one was gamification tools (20%). Generally, proportion of students who heard about all tools showed statistically significant increase after the intervention with the highest increase for gamification tools, which indicates the probable effectiveness of the intervention which increased it 235.9% after it was the least one before the intervention. A previous study reported that the most frequent tool was virtual patient learning (63.4%), with the least one also as gamification tools (21.2%) [19]. Gamification tools are emerging tools for medical study that are less known -as reported previously- which increase the engagement and interaction of students making educational content as games played by students. Students were excited regarding gamification tools after knowing about them after the intervention which was shown in their great feedback.

Chatbots are mainly used as a teaching assistant where it can answer students’ questions and suggest them resources. This provides an interactive tutor or a ward assistant in clinical practice helping students understand complex scenarios and develop decision-making skills [36].

Before intervention, electronic health records represented the most commonly reported AI tool in medical practice students previously heard about (51.3%), followed by robotic surgery (48.7%), while the least one was virtual assistants (32.8%). But after the intervention, medical imaging analysis was the most commonly reported tool (84.7%), followed by robotic surgery (84.1%), while the least one was predictive analytics (69.8%). All tools showed statistically significant increase after the intervention with the highest increase for virtual assistants (124.1%), followed by Clinical decision support system (121.2%). Khater et al. reported that the most frequent tool was medical imaging analysis (69.3%), followed by virtual assistants (51.7%) [19]. Virtual assistants help in managing appointments, provide personalized advice, improve patient outcomes, increase efficiency, streamline workflow, automate routine tasks, decrease costs, and save time of staff [37]. Moreover, medical imaging analysis was reported to accelerate diagnostic process, reduce errors as well as healthcare costs, and result in quicker diagnosis [38]. Therefore, it is important for medical students to be aware of those essential AI tools.

Regarding students’ knowledge and attitude towards AI and its application in medical education before the intervention, the statement with the highest agreement percent was “Believe that AI can improve the quality of medical education” (56.6% which increased to 74.1% after the intervention). Similarly, Khater et al. reported the same statement to have the highest agreement percent (89.5%) [19]. The statement with the highest improvement of agreement percent after intervention was “Familiar with the various AI tools available for educational purposes” (82.2%). This is a proof that the intervention was associated with an increase in the familiarity of medical students with the different AI tools used in medical education, while the statement with the lowest improvement of agreement percent was “You can use AI technologies for learning purposes confidently” (+ 18.7%). The discrepancy between the high increase in the knowledge or familiarity with the different tools and the low increase in their confidence in their ability to use can be due to the concise content in the educational intervention. The intervention was essentially designed to improve their knowledge about the different tools; however, it did not dive deeply into the details of how to use each tool in details as this needs a specific training for each tool. This means that students need a comprehensive program to increase their confidence in their ability to use AI for each tool after conducting need assessment for prioritization of the different tools.

Regarding students’ knowledge and attitude towards AI and its application in medical practice before the intervention, the statement with the highest agreement percent was “Willing to learn about the applications of AI in medicine” (58.7% which increased to 71.4% after the intervention). This indicates that the intervention was associated with an increase in the passion of students to learn more. Similarly, as in medical education, the statement with the highest improvement of agreement percent after intervention was “Familiar with the various AI tools used in medical practice” (116.2%), indicating the short term improvement of knowledge after the intervention; however, it had less impact on increasing their willing to be operated upon by an AI machine. Maybe, due to the recent nature with less confidence in the accuracy or safety of this technology where this confidence can be increased in the future after making sure of the evidence for its safety. Khater et al. reported that the statement with the highest agreement percent was “It is important for medical professional to understand how AI work” [19].

There was a strong positive significant correlation between medical students’ knowledge and attitude score in medical education and their score in medical practice both before and after the intervention. Moreover, the correlation was stronger after the intervention than before the intervention. This indicates that improving students’ knowledge in medical education is also associated with increasing their knowledge in medical practice.

### Study limitations

This study was a single-centered study, involving only the second year and the second internship year medical students. Although the sample size was calculated with a study power of 0.90, a multicentric study with a larger sample size would be better for generalization of results. Although this study was a prospective pre-post study; however, this design has a loss of follow-up bias (whose rate in this study was 14.1%). In addition, no control group was employed; therefore, it is difficult to prove that the intervention alone caused the improvement. The follow-up was also done after a short duration (only 2–3 weeks). Moreover, the outcomes were self-reported which may be biased. The authors acknowledge that knowledge and attitude are distinct constructs, therefore using a combined score may consolidate real differences between knowledge and attitude. Therefore, further research with a distinct construct is recommended.

### Study strengths

The study design of a prospective pre-post study has higher evidence than descriptive or retrospective studies. The study power was 0.90. Sampling method was stratified cluster random sample. The intervention was attractive, engaging, and concise as reported in the student’s feedback. It was associated with an increase in the familiarity of students about AI tools used in medical education and practice.

## Conclusion and recommendations

The intervention was associated with improved students’ knowledge and attitude towards AI and its application in both medical education and practice. Improving students’ knowledge in medical education is also likely associated with increasing their knowledge in medical practice. The intervention was also associated with an increase in the familiarity of medical students with the different AI tools used in both medical education and practice. However, students need a comprehensive program to increase their confidence in their ability to use AI. The intervention was also associated with an increase in the passion of students to learn more.

The most frequent preferred formats were small group discussion and workshops. The percent of students reporting the importance of learning programing and mathematics to comprehend the principles and uses of AI after the intervention was 88.9%. Therefore, it is recommended to make collaborative activities with other departments (mathematics, computer science) available for students desiring to participate in.

## Supplementary Information

Supplementary Material 1.

Supplementary Material 2.

## Data Availability

The datasets used and/or analysed during the current study are available from the corresponding author on reasonable request.

## Abbreviations

AI: Artificial intelligence
MFM-IRB: Institutional Research Board of Mansoura Faculty of Medicine
SD: Standard deviation
