# Supplementary material for: Medical students’ knowledge and attitude towards using artificial intelligence in medical education and practice: a pre-post study
Source: BMC Med Educ. 2026 Jun 23;26:1024. doi: 10.1186/s12909-026-09677-8 (PMC13288814; doi:10.1186/s12909-026-09677-8)
Supplement: Supplementary file 1 — Supplementary Material 1. [file 12909_2026_9677_MOESM1_ESM.pdf]

Dear Participants,

All answers will be kept confidential.

Demographic data

Phone number*: ------------

Faculty ID*:------------

E-mail*:------------

Technology background and use of AI in medicine

Do you think that learning programming or mathematics would help you better comprehend the principles and uses of artificial intelligence?

☐ 1. No

☐ 2. Yes

Choose AI tools in medical education and medical practice you previously heard about.		
**Considered an AI tool in medical education**	Yes	No
Personalized learning platforms		
Virtual patient learning		
Gamification tools		
Chatbots for student support		
Interactive smartboards		
Virtual reality simulation		
Automated exam grading systems		
**Considered an AI tool used in medical practice**		
Electronic health records		
Clinical decision support system		
Medical imaging analysis		
Personalized medicine		
Virtual assistants		
Robotic surgery		
Predictive analytics		
My preferred format for learning about artificial intelligence in medicine are (fill as many as appropriate)		
	Yes	No
Lectures		
Conferences		
Workshops		
Extracurricular activities		
Collaborative activities with other departments (mathematics, computer science)		

Knowledge and attitude towards AI and its application in medical education

	Strongly disagree	Disagree	Neutral	Agree	Strongly agree
Familiar with the concept of AI in education					
Familiar with the various AI tools available for educational purposes					
Believe that AI can improve the quality of medical education					
Believe that AI will revolutionize the way we learn in the future					
Willing to try out new AI technologies for medical educational purposes					
Confident in your ability to use AI technologies for learning purposes					
Comfortable with using AI in your education					
Comfortable with the idea of AI grading your academic work					
Concerned about the ethical implications of using AI in medical education					
Think "AI can aid in medical research"					
Think "it is important for universities to integrate AI technologies into their education					
Think "it is important for universities to provide training on how to use AI tool"					
Think "it is important for universities to teach students about the ethical implications of AI					
Think "AI can replace traditional teaching methods"					
Concerned about the potential for AI to replace human teachers					

Knowledge and attitude towards AI and its application in medical practice

	Strongly disagree	Disagree	Neutral	Strongly agree	Agree
Familiar with the various AI tools used in medical practice					
Willing to learn about the applications of AI in medicine					
Think "it is important for medical professionals to understand how AI works"					
Willing to use AI in your future medical practice					
Willing to be operated upon by an AI machine					
Think "AI can improve patient outcomes in Egypt"					
Believe that AI will replace human medical professionals in the future					
